# Self-Similarity and the Dynamics of Coarsening in Materials

**DOI:** 10.1038/s41598-018-36354-8

**Published:** 2018-12-18

**Authors:** Yue Sun, W. Beck Andrews, Katsuyo Thornton, Peter W. Voorhees

**Affiliations:** 1 Graduate Program in Applied Physics, Northwestern University, Evanston, IL 60208 USA; 2 Department of Materials Science and Engineering, Northwestern University, Evanston, IL 60208 USA; 3 Department of Materials Science and Engineering, University of Michigan, Ann Arbor, MI 48109 USA

## Abstract

Two-phase mixtures, from metallic alloys to islands on surfaces, undergo coarsening wherein the total interfacial area of the system decreases with time. Theory predicts that during coarsening the average size-scale of a two-phase mixture increases with time as *t*^1/3^ when the two-phase mixture is self-similar, or time independent when scaled by a time-dependent length. Here, we explain why this temporal power law is so robustly observed even when the microstructure is not self-similar. We show that there exists an upper limit to the length scales in the system that are kinetically active during coarsening, which we term the self-similar length scale. Length scales smaller than the self-similar length scale evolve, leading to the classical temporal power law for the coarsening dynamics of the system. Longer length scales are largely inactive, leading to a non-self-similar structure. This result holds for any two-phase mixture with a large distribution of morphological length scales.

## Introduction

Coarsening, also referred to as Ostwald ripening, occurs naturally in a wide array of materials, including metallic alloys^1,2^, polymers^3^, and semiconductors^4^. The classical theory of coarsening predicts that the microstructure of a two-phase system will evolve to be self-similar, or time independent when scaled by a time-dependent length. It also predicts that this length increases with time as *t*^1/3^ ^5,6^. The connection between the presence of both a self-similar structure and a temporal power law for the average length scale of the system is central in understanding the dynamics of coarsening processes, as emphasised by Mullins and Lifshitz, Slyozov and Wagner (LSW)^5–7^ and Onuki^8^. This connection has also been verified in simulations of systems of spherical particles^9–11^ and of bicontinuous microstructures where analytical solutions to the diffusion equation are not possible^12–15^. Experimentally, there are a few cases where self-similarity and temporal power laws have been observed^1,16^. However, in most cases a classical *t*^1/3^ power law for the average size scale of a two-phase mixture is observed without a self-similar two-phase morphology^17–23^. The most striking example is given by Marsh and Glicksman^21^ who show that even though a structure evolves in a non-self-similar fashion from a dendritic morphology to a polydisperse array of approximately spherical particles, the characteristic length scale of the two-phase system still increases as *t*^1/3^. Here, we use both time-resolved three-dimensional X-ray tomography and numerical simulations to demonstrate why coarsening microstructures can have a temporal power law for the average length scale while evolving in a non-self-similar manner.

## Results

### Experimental investigation

**Figure 1:**
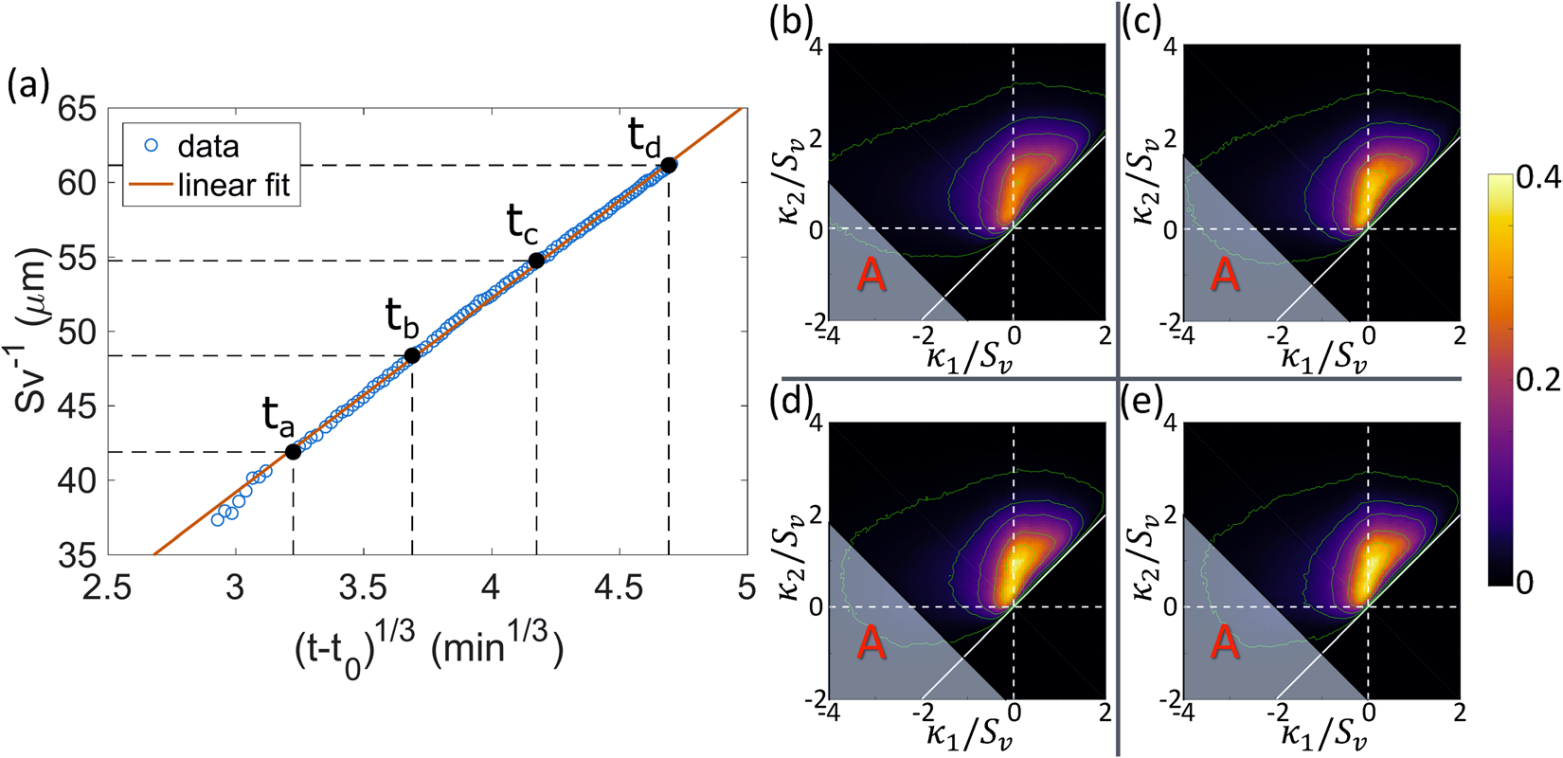
Quantitative characterisation of the evolution of the Al-Cu dendritic microstructure during coarsening. (**a**) Inverse of the specific interfacial area ${S}_{v}^{-1}$ versus shifted time to the one-third power (*t* − *t*_0_)^1/3^ from the experimental data (circles). The red line denotes the fitting of the data points using Equation 1. The fitting was carried out by performing a linear regression on experimental data of ${S}_{v}^{-3}$ against experimental time *t*. The four black dots show four times (*t*_*a*_ = 8.40 min, *t*_*b*_ = 25.12 min, *t*_*c*_ = 47.62 min, and *t*_*d*_ = 78.15 min) evenly spaced in ${S}_{v}^{-1}$. (**b**–**e**) Interfacial shape distributions (ISDs) at the four times marked in (**a**), respectively. The green lines are isovalue lines at 0.01, 0.05, 0.1, 0.2, and 0.3, outside-in. The shaded regions labelled as *A* on the ISDs show values in *κ*_1_ and *κ*_2_ that represent the 10% interfaces with the highest negative *H*.

We start by analysing a time-resolved X-ray Computed Tomography (XCT) three-dimensional dataset collected during an *in situ* experiment of isothermal dendritic microstructure coarsening in Al-Cu alloy^24,25^. See Methods for details. This system coarsens by interfacial energy driven diffusion of solute in the liquid phase. First, we test the growth of the specific interfacial area *S*_*v*_ against the temporal scaling law for the length scale of the system. We perform a linear regression on the specific interfacial area to the negative third power ${S}_{v}^{-3}$ against experimental time *t*, using a linear model given by

1 $${S}_{v}^{-3}(t)-{S}_{v}^{-3}({t}_{0})=kt,$$

where the cube of the initial length scale ${S}_{v}^{-3}({t}_{0})$ and the coarsening rate constant *k* are the fitting parameters. The result of the regression shows a high linear correlation between ${S}_{v}^{-3}$ and *t*, with an *R*^2^ of 0.9990. In Fig. 1a, we plot the result in the form of ${S}_{v}^{-1}$ versus (*t* − *t*_0_)^1/3^, where the intercept ${S}_{v}^{-3}({t}_{0})$ in the original fitting is converted to a shift in *t* with magnitude ${t}_{0}=-\,{S}_{v}^{-3}({t}_{0})/k$. It is clear that, except for a small deviation at the early stage of the experiment (*t* < 8.4 min), most of the data is well described by a straight line. This indicates that ${S}_{v}^{-1}\propto {(t-{t}_{0})}^{\mathrm{1/3}}$, or ${S}_{v}^{-1}\propto {t}^{\mathrm{1/3}}$ asymptotically. In other words, ${S}_{v}^{-1}$ in the dendritic system grows with the same temporal exponent as given in LSW theory.

**Figure 2:**
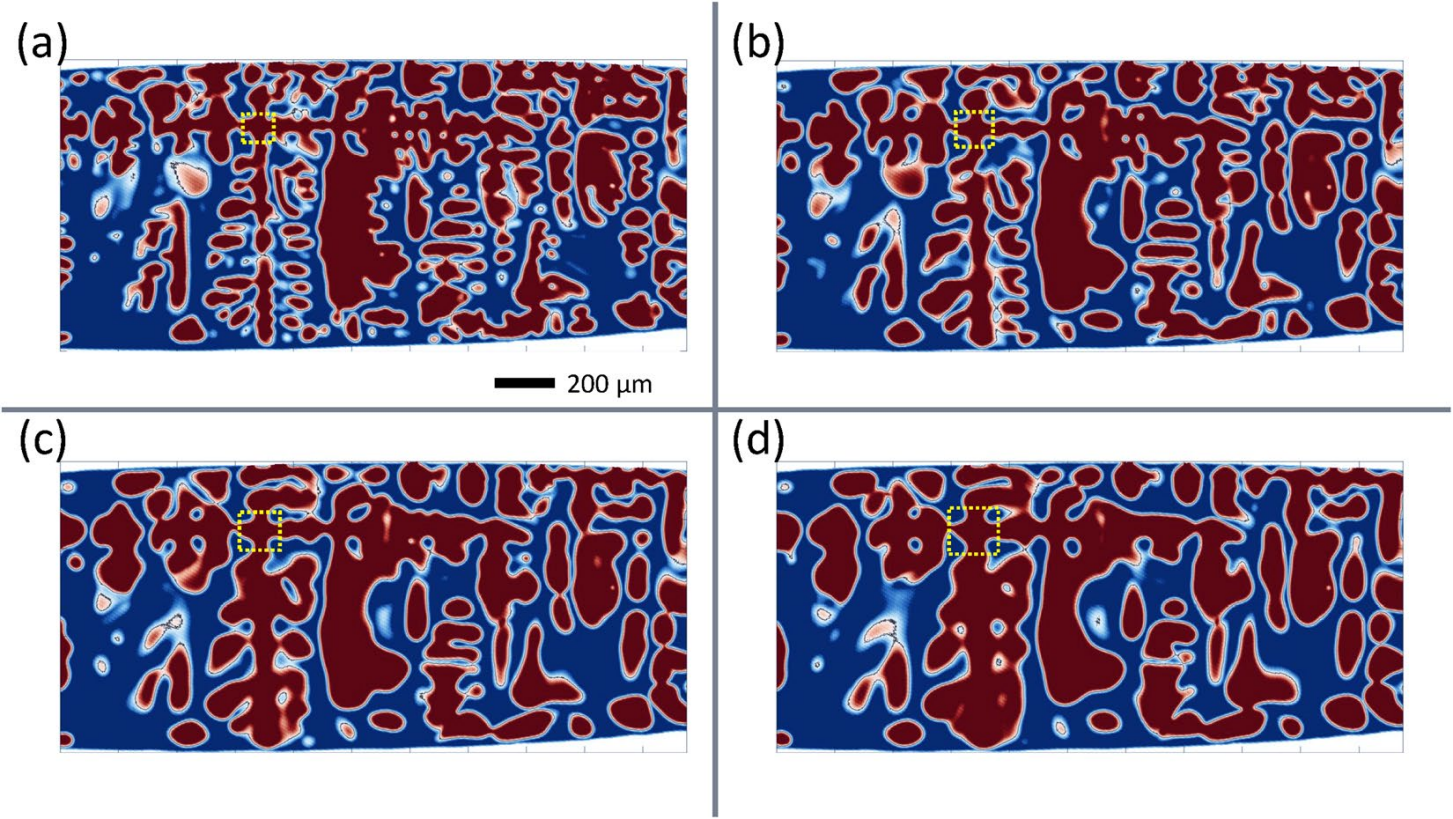
Cross-sections of the dendritic microstructure at four different times: (**a**) 8.40 min, (**b**) 25.12 min, (**c**) 47.62 min, and (**d**) 78.15 min.

However, despite this agreement, the morphology of the dendritic microstructure does not evolve in a self-similar manner during coarsening. Figure 2 shows the two-dimensional cross-sections of the microstructure at four different times. These times are chosen such that the change in ${S}_{v}^{-1}$ between adjacent times are the same (as shown in Fig. 1a). The cross-sections shown in Fig. 2 are made across the centres of the primary (horizontal direction) and secondary (vertical direction) dendrite arms. These plots show significant coarsening of the tertiary dendrite. On the other hand, the secondary dendrite arms, which are much larger than the tertiary arms, appear to maintain their overall shapes and positions during the experiment.

Quantitatively, the evolution of the Interfacial Shape Distribution (ISD)^26^ is not self-similar either. Figure 1b–e shows the ISDs of the solid-liquid interface at the same four times. An ISD gives the probability of finding a patch of interface with a given pair of principal curvatures (*κ*_1_, *κ*_2_), analogously to the particle size distribution for a system of spherical particles. The principal curvatures of each ISD are scaled by the *S*_*v*_ corresponding to the microstructure at that time. While the region with nonzero probability of the non-scaled ISDs becomes smaller with coarsening time, it remains approximately the same size for the scaled ISDs, which indicates the increase of length scales of the microstructure during coarsening. However, the ISDs do not scale in shape and intensity. More specifically, the distribution becomes more concentrated towards the *κ*_1_ = 0 line (vertical axis). This lack of self-similarity agrees with previous investigations^22^.

In order to elucidate the dynamics of this coarsening process, which is described by a *t*^1/3^ power law for the length scale yet is not self-similar, we calculate the two-point spatial correlation of the interfacial curvatures and analyse its temporal evolution. As is shown in a preceding study^25^, the two-point spatial correlation is capable of accurately and efficiently quantifying the spatial distribution of microstructural quantities in a statistical manner. By choosing an appropriate quantity of interest, the characteristic length scales of the dendritic microstructure can be robustly extracted.

**Figure 3:**
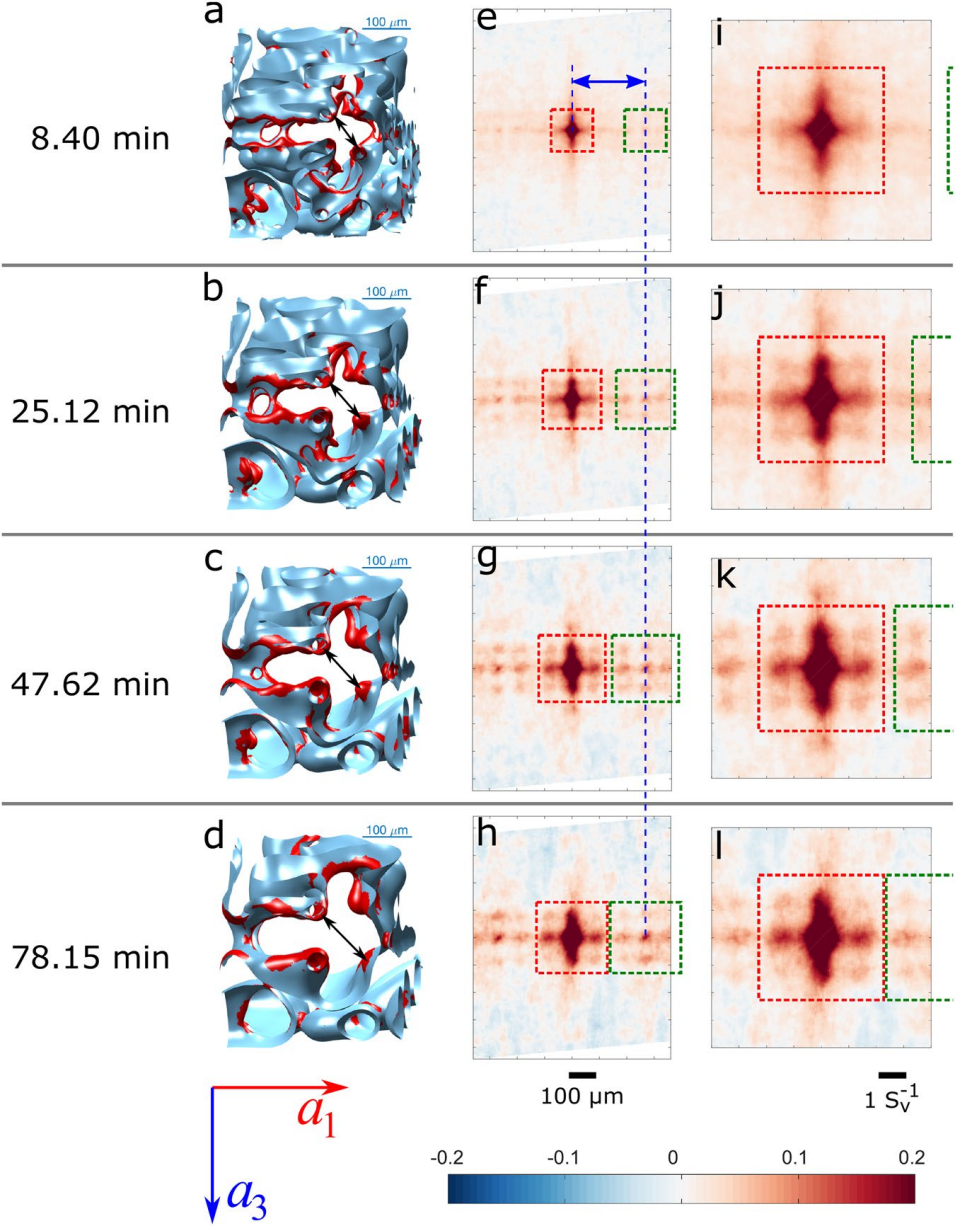
Illustration of the two-point Pearson auto-correlation of interfaces with 10% most negative mean curvature *H* at four representative times. (**a**–**d**) A subset of the sample interface showing the stem of a secondary dendrite arm in the region around the yellow dashed boxes in Fig. 2. (**e**–**h**) Slices of the Pearson auto-correlation map along a plane perpendicular to the secondary dendrite arms and across the origin. The horizontal and vertical axes are parallel to the primary dendrite growth (***a***_**1**_) and tertiary dendrite arm (***a***_**3**_) directions. (**i**–**l**) The same correlation maps as in (**e**–**h**), but scaled by ${S}_{v}^{-1}$.

As suggested by the Gibbs-Thomson equation, the evolution of the interface during coarsening is primarily controlled by the local interfacial mean curvature *H* = (*κ*_1_ + *κ*_2_)/2. Therefore, we use the interfacial mean curvature as the interfacial quantity of interest in the calculation of two-point spatial correlations. In ref.^25^, it is shown that interfaces with high negative mean curvatures are most spatially correlated among all interfaces in the dendritic microstructure. From the auto-correlation of these interfaces, characteristic length scales of the microstructure, including the secondary dendrite arm spacing *λ*_2_ and the secondary arm radius, can be extracted. Figure 3e–h shows the two-point auto-correlations of the 10% of interfacial patches with the most negative values of *H* at four different times. The correlations are presented in the form of Pearson correlation coefficients, which have values ranging from −1 to 1, indicating perfect anti-correlation (−1), no correlation (0), perfect correlation (1), or an intermediate state.

For visualisation, two-dimensional cross-sections of the two-point spatial correlations are shown, such that the horizontal and vertical axes align with the primary and tertiary dendrite arm directions, respectively, and the secondary dendrite arm direction is perpendicular to the paper plane. In all of the four plots, a four-fold lattice-like pattern is easily identified (outlined by the centre red dashed box in Fig. 3e–h). This four-fold pattern comes from the strong correlation of the troughs on the secondary dendrite arms in between the roots of the tertiary dendrite arms. These troughs are cylindrical interfaces with high negative curvature, which reside on the stems of the secondary dendrite arms. An example of this structure is marked by the yellow dashed boxes in Fig. 2. Enlarged 3-D views of the interface around this area are shown in Fig. 3a–d, in which the interfaces with the 10% most negative *H* are highlighted in red. From the region within the red dashed box at the centre of the correlation maps, it is clear that the “lattice” correlation pattern is expanding with coarsening time. This means that the secondary dendrite arms are coarsening in a way that the troughs on the secondary arms are moving away from each other.

To the left and right sides of the centre pattern, there are replicates of the centre “lattice” pattern (right side outlined by green dashed boxes in Fig. 3e–f), which come from correlations between adjacent secondary dendrite arms. The presence of these second-order correlation peaks is evidence for the periodicity of the structure along the primary dendrite growth direction. In other words, the secondary dendrite arms are well-aligned and equally spaced. The evolution of these second-order lattice-like patterns exactly mirrors the expansion of the primary pattern at the centre. However, the distance between the second- and first-order patterns remains constant across time, as shown by the blue dashed lines in Fig. 3e–h. This indicates that the secondary dendrite arm spacing *λ*_2_ is not changing with time.

In Fig. 3i–l, the correlation maps in Fig. 3e–h are scaled by ${S}_{v}^{-1}$ at each time. In the scaled plots, the correlation pattern within the red dashed box remains constant (except for the decreased signal intensity in the earliest time plot due to a high level of noise). Since we have already shown in Fig. 1a that ${S}_{v}^{-1}$ scales with (*t* − *t*_0_)^1/3^, this indicates that the microstructures associated with the correlation pattern within the red dashed box (i.e., the secondary dendrite arm trunks) are coarsening following the *t*^1/3^ temporal scaling law in the LSW theory. In the green dashed box to the right of the centre red box, the size of the second-order correlation pattern also remains constant in the scaled correlation plots. However, the second-order patterns on both sides are moving towards the centre of the correlation map in these scaled plots.

Marsh and Glicksman conjectured that length scales larger than a certain limit will be “kinetically inactive” during coarsening^21^. Using the measurements of the spatial correlations shown in Fig. 3, we have shown that such kinetically inactive length scales in fact exist. We find that length scales smaller than the self-similar length scale grow self-similarly and in proportion to *t*^1/3^, which agrees with LSW theory. On the other hand, length scales larger than the self-similar length scale remain largely inactive, until the self-similar length scale, which follows the same *t*^1/3^ temporal scaling law, outgrows those length scales, thus making them kinetically active. It is only through the two-point correlation functions that it is possible to statistically quantify the relevant length scales in microstructures with complicated morphologies. By tracking the evolution of these length scales through time, we have determined the self-similar length scale that differentiates kinetically active and inactive length scales in the dendritic microstructure during coarsening.

### Computational validation

To examine the existence of inactive length scales in coarsening and to illustrate that the conclusions are not limited to dendritic structures, we simulate coarsening of a simpler, two-dimensional microstructure containing second-phase particles with a bimodal particle size distribution. The two modes of this distribution correspond to populations of large and small particles, where the difference in radii is sufficiently large such that the large particles may be inactive. Comparing the overall evolution of this system to the evolution of each population of particles should test the hypothesis of inactive length scales. Details regarding the generation of the initial structure are provided in Methods.

**Figure 4:**
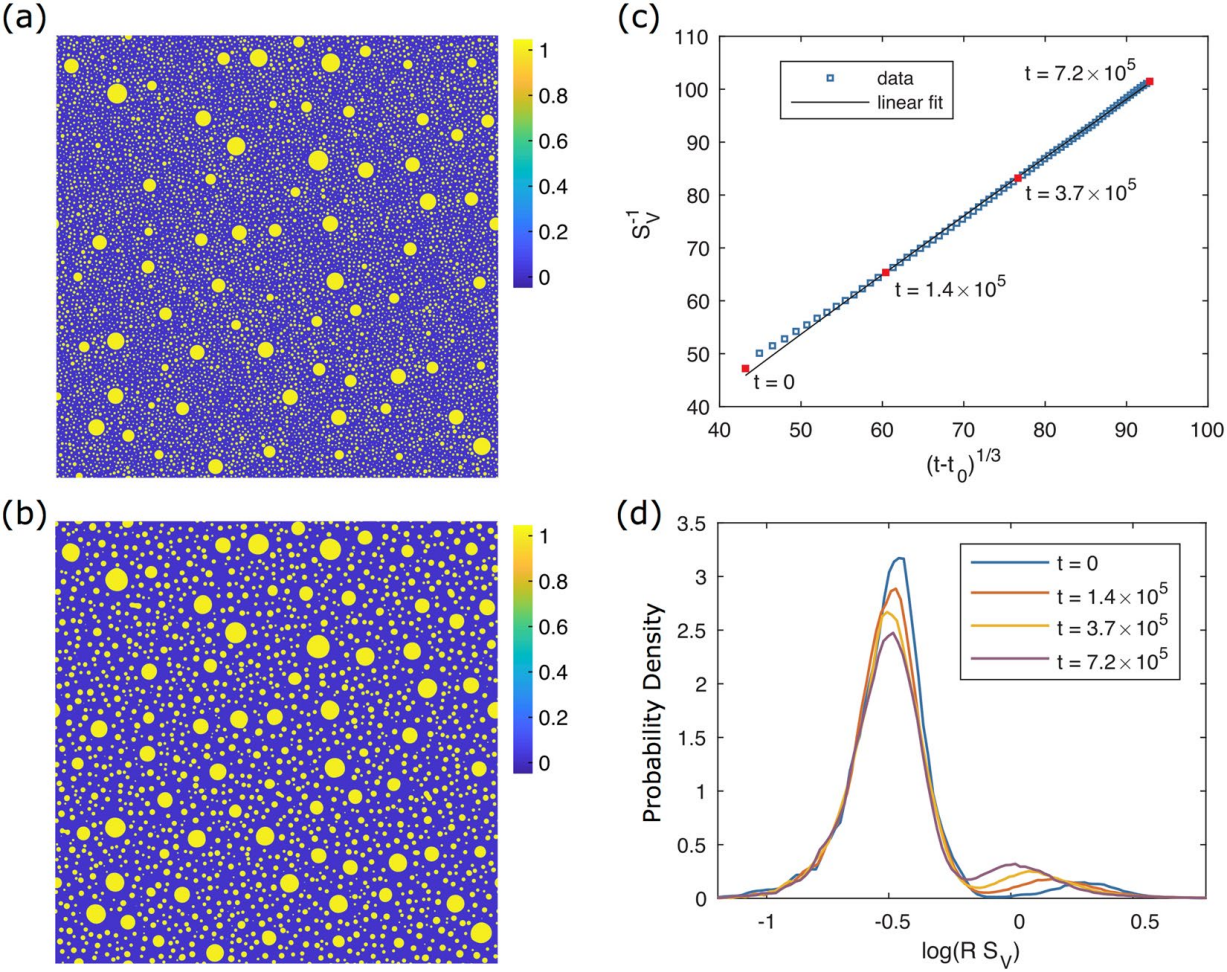
Illustrations of the 2-D phase field simulation. (**a**) Initial concentration field (*t* = 0, ${S}_{v}^{-1}=47.2$) containing 5% volume fraction of large particles and 16% of small particles. (**b**) Final concentration field (*t* = 7.2 × 10^5^, ${S}_{v}^{-1}=101.5$). (**c**) Evolution of characteristic length ${S}_{v}^{-1}$ vs. time *t* plotted alongside a fit to the expected coarsening power law. (**d**) Interfacial radius-of-curvature distribution weighted by interfacial length at the times indicated in (**c**). Two peaks are present corresponding to the small and large particle distributions introduced in the initial condition.

Coarsening of the structure was simulated using the Cahn-Hilliard equation^27,28^ with periodic boundary conditions. The initial and final microstructures are shown in Fig. 4a,b. The large particles have grown at the expense of their smaller neighbours, and the small particles have coarsened, either growing or shrinking and disappearing. The characteristic length of the system in terms of ${S}_{v}^{-1}$ has increased from 47.2 to 101.5. The temporal evolution of the morphology is described quantitatively in Fig. 4c,d. While some deviation is apparent at early times, we find excellent agreement overall between the evolution of characteristic length and the expected coarsening power law given by Equation 1. This fit is shown in Fig. 4c with ${t}_{0}=-{S}_{v}^{-3}({t}_{0})/k$, and its coefficient of determination is *R*^2^ = 0.9995.

Figure 4d depicts distributions of interfacial radius of curvature (i.e., the reciprocal of scalar curvature), scaled by the characteristic length ${S}_{v}^{-1}$. These distributions are weighted by interfacial length and normalised. Therefore, they represent the probability that a point on the interface has a particular radius of curvature. These distributions would be equivalent to one-dimensional ISDs, except that they are functions of radius of curvature rather than curvature itself. For visualisation, the distributions are plotted in semilog scale, where log is taken for the horizontal axis. These choices (independent variable, weighting, and axis scale) were made to clearly represent the two populations with highly disparate length scales.

Two peaks are present in Fig. 4d, corresponding to large and small particles. Over time, the small-particle peak remains stationary about $R=0.34\,{S}_{v}^{-1}$, indicating that the small particles coarsen with the same rate as the overall structure. The height and area of the large-particle peak increase at the expense of the small-particle peak, which means that it contains a growing proportion of the total interfacial length in the system. In addition, the large-particle peak shifts leftward, indicating that the large particles are coarsening more slowly than the characteristic length scale of the system. These trends are confirmed when we examine the unscaled behavior: the location of the large-particle peak shifts from *R* = 94 to *R* = 106, or by 12%, over the course of the simulation, while the small-particle peak shifts from *R* = 17 to *R* = 34, or by 100%. Thus the change in the small-particle peak is much larger relative to its initial state, which explains why it dominates the evolution of the overall system. These simulation results support the hypothesis that there exists a critical length scale that separates populations of interfaces that evolve self-similarly from those that do not, even though the simulated microstructure is vastly different from its experimental counterpart. We also identify that the domination of the actively evolving population is responsible for the *t*^1/3^ growth power law observed so universally.

## Discussion

Using two-point correlation functions, time resolved X-ray tomography, and simulation, we show that it is possible to statistically quantify the wide range of length scales present in microstructures with complicated morphologies. We find that in a system with a multimodal distribution of morphological feature sizes, active coarsening only occurs with microstructure features smaller than the self-similar length scale, which delimits the smallest length scale that remains largely inactive. Length scales below the self-similar length scale will coarsen in a self-similar manner, following the classical *t*^1/3^ temporal power law. Features with larger length scales do not actively participate in the coarsening process. Therefore, the interfacial morphology of the entire structure is not self-similar, while still exhibiting classical temporal power laws for the coarsening process. These larger features are never completely inactive, as we have shown; they simply evolve at much slower rates and thus are essentially stationary over the time scale of coarsening of the smaller features. When the self-similar length scale approaches these formerly larger length scales, they become kinetically active. However, this does not suggest that the coarsening of these features suddenly becomes faster; they will instead coarsen at a rate consistent with their length scale. Thus, the change from inactive to active will be gradual, as shown in the many experimental results of coarsening in systems with a large range of length scales. This mechanism is applicable to any two-phase mixture exhibiting a large range of length scales that undergoes coarsening.

## Methods

### Experimental

The sample used in the experiment was made from a directionally solidified Al-Cu alloy with 19 wt% of Cu. The raw material was cut into a cylinder that is 5 mm length by 1 mm diameter, with the axial direction approximately parallel to the direction of solidification. During the experiment, the sample was heated to 558 °C (5 °C above the eutectic temperature), forming a liquid-solid mixture with a constant amount of the two phases with volume fraction of liquid equal to 47.04%. The sample was then held at that temperature to coarsen for 79 minutes while being scanned by X-ray computed tomography (XCT). The XCT data was then reconstructed into 3-D views at a temporal rate of 44 *s* per view and with a voxel size of (1.79 *μ*m)^3^. The full experimental dataset consists of 94 time frames, with the inverse of the specific interfacial area ${S}_{v}^{-1}$ ranging from 37.931 *μ*m (first time frame) to 61.263 *μ*m (last time frame).

A voxelised representation of the interface was used to calculate the two-point spatial correlations of the interfacial mean curvature *H*, which results in an interface with thickness of one voxel length. Each interfacial voxel is associated with an interfacial mean curvature value *H*, which was calculated using the signed distance function with respect to the interface. The population of interfacial voxels was split into deciles based on their values of *H*. Two-point Pearson auto-correlations of the interfacial voxels within each decile were calculated, using only the interfacial voxels (as opposed to all voxels in the sample bulk) as the normalisation. See ref.^25^ for details.

### Computational

Particle coarsening was simulated using the Cahn-Hilliard equation^27,28^:

2 $$\frac{\partial c}{\partial t}=\nabla M\cdot \nabla (\frac{\partial f(c)}{\partial c}-{\varepsilon }^{2}{\nabla }^{2}c),$$

where the bulk free energy *f*(*c*) is given by the double well potential $f(c)=\frac{W}{4}{c}^{2}{\mathrm{(1}-c)}^{2}$, with well height parameter *W* = 0.4, and the mobility *M* and gradient energy coefficient *ε* are scalar constants, *M* = 1 and $\varepsilon =\sqrt{0.2}$. The size of the simulation domain was 6400^2^ and periodic boundary conditions were enforced. The domain was discretised by a uniform grid with Δ*x* = 1, which results in 3–5 grid points through the interface as defined by $c\in [0.1,0.9]$. Explicit (forward Euler) time integration was used with a time step of Δ*t* = 0.05. These simulation parameters are dimensionless, and the simulation results are therefore nondimensional, in contrast to the experimental results.

The structure used as an initial condition for the simulation was generated in stages. Starting from a uniformly zero concentration field, large particles were generated sequentially until $\bar{c}=0.051$. Then small particles were generated until $\bar{c}=0.210$, and finally a uniform concentration field was added to approximate the mean field concentration during coarsening, resulting in $\bar{c}=0.218$. Details of these stages are given below.

To generate a particle, three random floating-point numbers were sampled from a uniform distribution over [0, 1]. The first two were multiplied by the domain length to find the coordinates of the particle centre, *x*_0_ and *y*_0_. The remaining number was interpolated onto a discrete cumulative particle size distribution to determine particle radius *R*. To prevent the creation of overlapping particles, the numbers were discarded if *c*(*x*, *y*) > 0.5 within a fixed radius of (*x*_0_, *y*_0_). This radius, *r*_contact_, corresponds to the minimum allowed distance between the centre of the new particle and the perimeter of any existing particles. For large particles, *r*_contact_ = 384 was used, and for small particles, *r*_contact_ = 32. If this check was passed, then a new particle was added to the existing concentration field *c*_0_(*x*, *y*):

3 $$c(x,y)={c}_{0}(x,y)+\frac{1}{2}(1+\,\tanh \{[R-\sqrt{{(x-{x}_{0})}^{2}+{(y-{y}_{0})}^{2}}]/2\}).$$

If the point (*x*_0_, *y*_0_) was close enough to one or more domain boundaries, images of the particle would also be added at the opposing boundaries to ensure periodicity.

**Figure 5:**
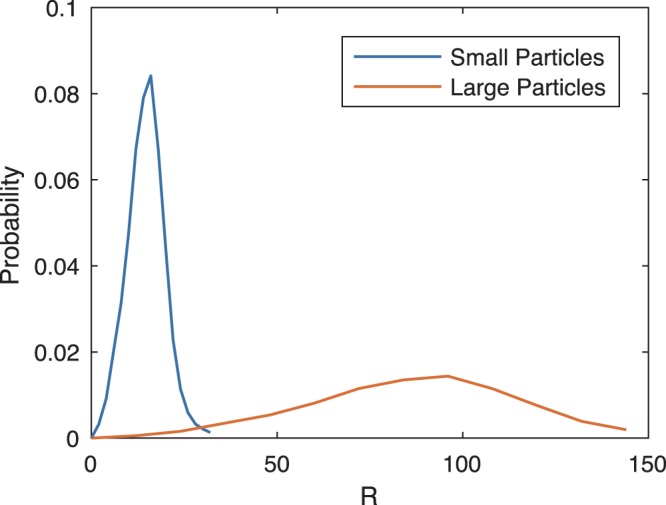
Particle size distributions sampled to generate the simulation initial condition. They are normalised independently, and so do not reflect probability in the final structure, where large particles would be much less frequent.

To minimise morphological evolution within each mode, the particle size distributions (PSDs) used for sampling were based on the steady-state PSD for $\bar{c}\approx 0.22$. This PSD was obtained from a smaller 1600^2^ phase field simulation with $\bar{c}=0.221$ initialised with a single-modal PSD with average radius $\bar{R}=8$. The PSD of the single-modal simulation appeared to converge after *t* = 5 × 10^4^, and so the steady state PSD was taken to be the time average over 6 × 10^4^ ≤ *t* ≤ 10^5^. The generating PSDs are shown in Fig. 5. They reflect input $\bar{R}$ values of 16 for small particles and 96 for large particles. Due to truncation of the steady state PSD at $R/\bar{R}=2$ and $R/\bar{R}=1.5$ for small and large particles, respectively, these result in actual average radii of 14.6 and 80.9.

Finally, the mean field concentration due to the Gibbs-Thomson effect was added uniformly to the concentration field. This was approximated as a rule of mixtures using the input average radii for the distributions,

4 $${\rm{\Delta }}c\approx \frac{\lambda }{96}{V}_{L}+\frac{\lambda }{16}{V}_{S}=0.0083,$$

where the volume fractions *V*_*L*_ and *V*_*S*_ of large and small particles are 0.24 and 0.76, respectively, and the capillary length *λ* is given in terms of the phase field parameters as

5 $$\lambda =\frac{\frac{1}{6}\varepsilon \sqrt{\frac{W}{2}}}{\frac{1}{2}W}=\frac{1}{6}.$$

The final average composition after this addition was $\bar{c}=0.218$.

Scalar curvature (and by extension radius of curvature) and characteristic length ${S}_{v}^{-1}$ were calculated by stacking together two copies of the 6400^2^ structure in the out-of-plane direction, extending the structure into 3-D. This allowed use of the procedure from Park *et al*.^29^, with scalar curvature equal to twice the calculated mean curvature.

## Data Availability

The experimental datasets generated during and/or analysed during the current study are available from the corresponding author on reasonable request. Simulation datasets will be published at Materials Commons (https://materialscommons.org/).
